# Identification and Compensation Technique of Non-Uniform Temperature Field for Lamb Wave-and Multiple Sensors-Based Damage Detection

**DOI:** 10.3390/s19132930

**Published:** 2019-07-02

**Authors:** Hu Sun, Junyan Yi, Yu Xu, Yishou Wang, Xinlin Qing

**Affiliations:** School of Aerospace Engineering, Xiamen University, Xiamen 361005, China

**Keywords:** structural health monitoring, Lamb wave, temperature field identification, temperature compensation, non-uniform temperature field

## Abstract

Lamb wave-based damage detection for large-scale composites is one of the most prosperous structural health monitoring technologies for aircraft structures. However, the temperature has a significant effect on the amplitude and phase of the Lamb wave signal so that temperature compensation is always the focus problem. Especially, it is difficult to identify the damage in the aircraft structures when the temperature is not uniform. In this paper, a compensation method for Lamb wave-based damage detection within a non-uniform temperature field is proposed. Hilbert transform and Levenberg-Marquardt optimization algorithm are developed to extract the amplitude and phase variation caused by the change of temperature, which is used to establish a data-driven model for reconstructing the reference signal at a certain temperature. In the temperature compensation process, the current Lamb wave signal of each exciting-sensing path under the estimated structural condition is substituted into the data-driven model to identify an interpolated initial temperature field, which is further processed by an outlier removing algorithm to eliminate the effect of damage and get the actual non-uniform temperature field. Temperature compensation can be achieved by reconstructing the reference signals within the identified non-uniform temperature field, which are used to compare with the current acquired signals for damage imaging. Both simulation and experiment were conducted to verify the feasibility and effectiveness of the proposed non-uniform temperature field identification and compensation technique for Lamb wave-based structural health monitoring.

## 1. Introduction

Lamb wave-based structural health monitoring (SHM) technique has been proved to be one of the strongest techniques for online and real-time damage detection of aircraft structures because of the superior capability of travelling long distances and covering large monitoring area with limited numbers of piezoelectric sensors [1]. Plenty of Lamb wave-based damage detection algorithms, including delay and sum [2,3], phased array [4,5], tomography [6,7,8,9,10,11], and migration [12,13], have been developed. Comprehensive reviews on the application of Lamb wave for structural health monitoring can be found in the literature [1,14,15]. Depending on whether reference data of Lamb wave in the pristine structure is needed or not, these algorithms can be divided into two categories, i.e., reference-free algorithms [16,17,18,19] and reference-dependent ones [2,3,4,5,6,7,8,9,10,11,12,13]. Reference-dependent algorithms are more practical than reference-free ones for real application in aircraft structures due to the complex nature of the Lamb wave, including multiple mode, modes conversion and scattering at the boundary.

The principle of reference-dependent algorithms is to subtract the reference data from the current data collected in the current unknown state, which makes the difference data only contain the information of damages. However, Lamb wave is vulnerable to time-varying environmental parameters, including temperature, humidity, load, and so on. Temperature shows a significant influence on the amplitude and phase of Lamb wave signals, which is even larger than that resulting from damages. As a result, temperature compensation is often needed for accurate damage identification.

Some temperature compensation technologies have been developed. Lu and Michaels [20] introduced a baseline signal stretch (BSS) compensation method. The method needs to record a set of reference signals at different temperatures, and the signal closest to the current temperature is adjusted to best match the current signal by time dilation or compression. Konstantinidis, et al. [21] proposed a temperature compensation technology termed optimal baseline subtraction (OBS) to reduce the effects of temperature on the long-term stability. However, in real applications, OBS requires a large reference database and BBS is constrained by mode purity. Clarke, et al. [22] reduced the signals required in the database by combining BBS and OBS. The signal is stretched in the frequency domain so as to keep the frequency content of the original signal. Roy, et al. [23] developed a physics-based temperature compensation model in which the influence of temperature on physical properties of PZT, adhesive and host structure is all considered, and a linear system identification model is then introduced to reconstruct signals by relating the temperature and the signal features. Wang, et al. [24] proposed a method combining OBS with an adaptive linear neuron(ADALINE)-based filtering method. Fendzi, et al. [25] presented a data-driven temperature compensation model which is expressed as a linear relationship between temperature and variation of wave amplitude (phase).

All the above temperature compensation algorithms perform well in the uniform temperature field. However, during the actual flight of an aircraft, the structural temperature field is usually non-uniform [26]. Moreover, the temperature difference at different points in the structure may be as large as dozens of degrees due to the illumination angle of sunlight, the local overheating, etc. In this case, it is costly to get the structural temperature field by arranging an additional dense temperature sensor array.

However, in order to identify the damage, a sufficient number of piezoelectric sensors are arranged in the structure. If assuming that the temperature field of the structure changes continuously and there is only a small amount of damage in the structure, it is possible to identify the structural temperature field through the Lamb wave variation of each exciting-sensing path and then perform temperature compensation for damage detection, which is not realized by additional temperature sensors but necessary piezoelectric sensors.

A data-driven temperature compensation algorithm for Lamb wave-based damage detection in a non-uniform structural temperature field is proposed. Parameter estimation algorithms containing Hilbert transform and Levenberg-Marquardt optimization are employed to extract variations of wave amplitude and phase of pre-acquired Lamb wave signals for a data-driven model, and reconstruct wave signals at a given temperature at the estimated condition. Variations of wave amplitude and phase under different uniform temperature fields are used to establish a data-driven model. The process of the proposed temperature compensation algorithms contains two steps. The first step is to identify the non-uniform temperature field by extracting the variations of Lamb wave of each exciting-sensing paths and eliminate the effect of damage by an outlier removing method. The second step is to reconstruct Lamb wave signals under the estimated temperature field in the pristine structure. Both finite element simulation and experiment are employed to verify the feasibility of the proposed method.

This paper is organized as follows: In Section 2, principle and procedure about temperature compensation are described. Simulated and experimental results are presented in Section 3 and Section 4, respectively. Section 5 concludes the work that has been done in this paper and discusses some problems that can be further studied.

## 2. Temperature Compensation Principle

### 2.1. Parameter Estimation and Signal Reconstruction for Compensation

**Figure 1:**
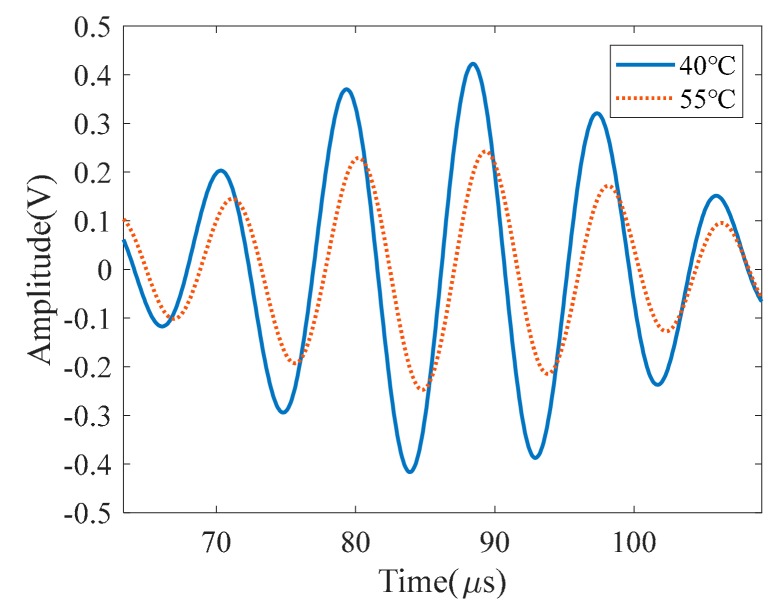
Typical signal differences under different temperatures.

For reference-dependent Lamb wave-based SHM, the influence of the damage on Lamb wave signal can be obtained by subtracting the reference signal acquired in the undamaged structure from the current signal collected in the damaged structure at the same temperature. However, signals collected by piezoelectric sensors are easily affected by temperature, which is manifested in the amplitude variation and the phase shift. As shown in Figure 1, wave amplitude at 55 °C is smaller than that at 40 °C, while the phase is delayed. The change of wave amplitude and phase caused by temperature variation is so significant that it may hide the damage information. Consequently, temperature compensation is needed. In this paper, the changes of wave amplitude and phase resulting from the change of temperature are quantitatively characterized to establish the relation between Lamb wave signal and temperature.

The relationship between the signal $y_{RT}(t)$ at the reference temperature *RT* and signal $y_{T}(t)$ at the temperature *T* can be assumed as

(1) $$y_{T}(t)=A(T)y_{RT}(s(T)t)$$

where A(T) is a temperature-dependent constant called the amplitude factor which is used to adjust the amplitude, while s(T) is termed as the scale factor used to adjust the phase. The complex form of a real signal can give the information for both the amplitude and the instantaneous phase. The complex form c(t) of the real signal y(t) is defined as

(2) $$c(t)=y(t)+j\hat{y}(t)$$

(3) $$\hat{y}(t)=H[y(t)]=\frac{1}{\pi }\int_{-\infty }^{+\infty }\frac{y(\tau )}{t-\tau }d\tau$$

where $\hat{y}(t)$ is the Hilbert transform of the real signal y(t). Equation (1) can be replaced with

(4) $$c_{T}(t)=A(T)c_{RT}(t)e^{j(P(T))}$$

Here, $c_{T}(t)$ is the complex form of $y_{T}(t)$, and P(T) is the phase factor. The estimated real signal $y_{T}^{E}(t)$ can be reconstructed from its complex form as

(5) yTE(t)=Re(cT(t))

where Re(•) denotes the real part.

Thus, the next problem that needs to be solved is to estimate these two parameters A(T) and P(T). The LM algorithm, which is an effective optimization algorithm solving the problem of parameter redundancy by combining both Newton’s method and gradient descent method, is employed to estimate these two parameters. The LM algorithm, in general, is a process of iteration, thus the iteration step size plays a major role in finding the best solution, defined as Equation (6).

(6) $$\begin{array}{l} \theta =\arg\min\sum_{i=1}^{N}r_{i}{}^{2}\\ r(i)=y_{T}(t_{i})-y_{T}^{E}(t_{i}) \end{array}$$

where $\theta ={\left[A(T),P(T)\right]}^{T}$ and $y_{T}^{E}(t_{i})$ is the reconstructed current signal. The parameter estimation takes the signals at the current temperature *T* and the reference temperature RT as inputs, and takes the amplitude factor *A* and the phase factor *P* as outputs. The iteration process of Newton’s method can be described as following equations.

(7) $$\begin{array}{l} \theta_{k+1}=\theta_{k}-H^{-1}g\\ g_{j}=2\sum_{i=1}^{N}r_{i}\frac{\partial r_{i}}{\partial \theta_{j}}\\ H_{jk}=2\sum_{i=1}^{N}(\frac{\partial r_{i}}{\partial \theta_{j}}\frac{\partial r_{i}}{\partial \theta_{k}}+r_{i}\frac{\partial^{2}r_{i}}{\partial \theta_{j}\partial \theta_{k}}),J_{ij}=\frac{\partial r_{i}}{\partial \theta_{j}} \end{array}$$

where $g_{j}$ represents the $j^{th}$ parameter’s gradient, J is the Jacobian matrix, and H is the Hessian matrix. In Gauss-newton method, to reduce the computational burden, the Hessian matrix is simplified as $H\approx 2J^{T}J$ with the assumption that the second part of the Hessian matrix in Equation (7) can be removed when $r_{i}$ is small. The LM algorithm introduces an identity matrix into the H to avoid the problem that a matrix is not invertible. Thus, the step size of the LM algorithm is represented as Equation (8).

(8) $$\theta_{k+1}=\theta_{k}-(J^{T}J+\mu I^{-1})J^{T}r.$$

where *μ*, called the damping factor, is used to control the iteration step size. When the value of the error formula becomes smaller, the value of *μ* is decreased by dividing by the given damping factor coefficient *λ*, and the value of *μ* is increased by multiplying by *λ* when the calculated error becomes larger. Though *μ* is automatically updated according to the error of every iteration result, *λ* determines the final accuracy and the iterations. In this paper, the initial values of *μ* and *λ* are set at 0.01 and 10 respectively.

If the parameter estimation is applied to all baseline signals at different temperatures, there are amplitude and phase factors with respect to temperature *T*. Therefore, a data-driven model relating *θ* and *T* can be established by a regression function and the signal at any temperature can be reconstructed by using Equations (4) and (5) as long as the current temperature is known.

### 2.2. Identification and Compensation of Non-Uniform Temperature Field

In this section, the process of temperature field identification and compensation will be introduced in detail.

**Figure 2:**
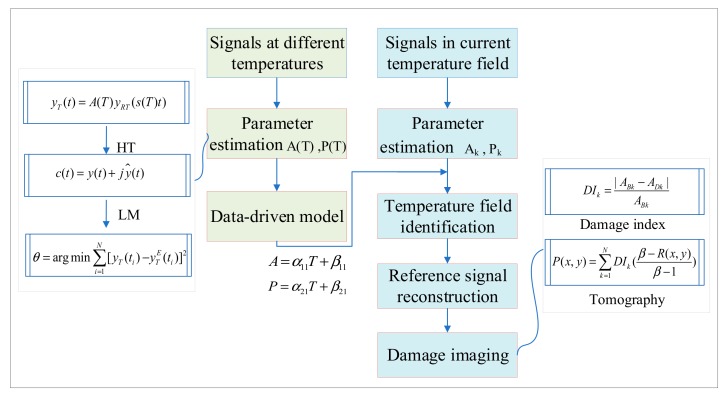
Process of temperature compensation for damage detection.

As shown in Figure 2, temperature compensation contains two parts, i.e., the training data-driven model and identifying the current temperature field for damage detection. When training the data-driven model, the pristine structure is placed in a space with adjustable uniform temperature. A set of Lamb wave signals are acquired under different temperatures and substituted to the parameter estimation algorithm to get a data-driven model relating θ and *T*. When identifying the temperature field for damage detection, Lamb wave signals of all exciting-sensing paths are collected from the current structure with an unknown temperature field. The parameter estimation algorithm is used to identify and define the temperatures along all exciting-sensing paths, which form the current non-uniform temperature field.

However, the current structure may be damaged. Other than temperature, damage also induces changes of Lamb wave signals in the amplitude and the phase. Therefore, the estimated temperature of the exciting-sensing path in the damaged region could be different from the actual temperature. Namely, the temperature values of paths affected by damages do not conform to the overall trend of temperature-trained Lamb wave data in the pristine structure. An additional process is needed to modify the temperature in the damaged region.

**Figure 3:**
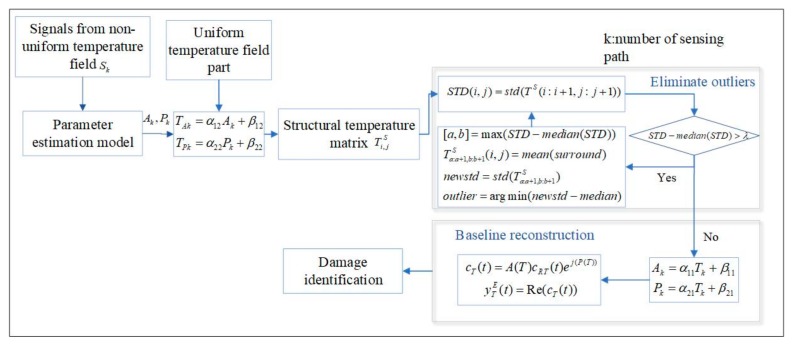
The detailed process of temperature field identification and compensation.

As shown in Figure 3, the structural temperature field is constructed by taking the temperature value of each exciting-sensing path as the pixel value of the path midpoint. Assuming the actual temperature field changes evenly, extreme points only occur at the damaged location. An outlier removing algorithm, which is an iterative optimization algorithm, is used to eliminate the outlier and reconstruct the actual temperature field. In the algorithm, a standard deviation matrix is built first by calculating the standard deviation of every two-dimensional matrix which consists of adjacent elements of the original temperature matrix. The standard deviation matrix is subtracted by the median of the matrix later and a threshold is used to find the zone that is effected by the damage. The elements of the identified zone are replaced by the mean of the surrounding elements one by one to calculate a new standard deviation. Finally, the element with the new standard deviation closest to the median of the matrix is removed and replaced.

As long as the temperature field is identified, reference signals can be reconstructed by Equation (4). The damage imaging algorithm is subsequently complemented with current signals and reconstructed reference signals.

In this paper, Lamb wave-based tomography [6] is applied to identify the location of the damage. Specific formulas are shown in the following equations

(9) $$DI_{k}=\frac{\left|A_{Bk}-A_{Dk}\right|}{A_{Bk}}$$

(10) $$P(x,y)=\sum_{k=1}^{N}P_{k}(x,y)=\sum_{k=1}^{N}DI_{k}(\frac{\beta -R_{k}}{\beta -1})$$

where $DI_{k}$ is the damage index of the $k^{th}$ sensing path, and $A_{Bk}$ and $A_{Dk}$ refer to the maximum amplitude of the reconstructed reference signal and the current signal, respectively. P(x,y) is the probability of damage at position (x,y). β is a scaling parameter, which decides the influencing area of each sensing path [7]. Therefore, the determination of its value is related to the placement of the sensors. In order to get a more accurate damage location in the case of the sensor setting in this paper, β is set at 1.45 [7]. $R_{k}$ is defined as

(11) $$R_{k}=\left\{\begin{array}{c} RD_{k},RD_{k}<\beta \\ \beta ,RD_{k}>\beta \end{array}\right.$$

where

(12) $$RD_{k}=\frac{\sqrt{{\left(x-x_{ak}\right)}^{2}+{\left(y-y_{ak}\right)}^{2}}+\sqrt{{\left(x-x_{ak}\right)}^{2}+{\left(y-y_{ak}\right)}^{2}}}{\sqrt{{\left(x_{ak}-x_{sk}\right)}^{2}+\sqrt{{\left(y_{ak}-y_{sk}\right)}^{2}}}}$$

$\left(x_{ak},y_{ak}\right)$ and $\left(x_{sk},y_{sk}\right)$ are the coordinates of the transmitter and the receiver of the *k*th exciting-sensing path. If the damage is just on the exciting-sensing path, $R_{k}=0$, $P_{k}(x,y)=1$. If $R_{k}=\beta$, $P_{k}(x,y)=0$, which indicates that the point (x,y) is already outside the scope of the path defined by β. P(x,y) of the damaged area will be larger than the other points by accumulating probability.

## 3. Simulation Verification

### 3.1. Parameter Estimation and Signal Reconstruction

In this paper, the finite element method (FEM) is employed to simulate the effects of temperature on Lamb wave signals by changing temperature-sensitive material parameters, including Young’s modulus of the monitored structure, shear modulus of adhesive, the piezoelectric constant (d31), and permittivity constant (e33) of the piezoelectric sensor. Material parameters in Table 1 are chosen to be temperature-dependent parameters and used in the simulation model.

**Figure 4:**
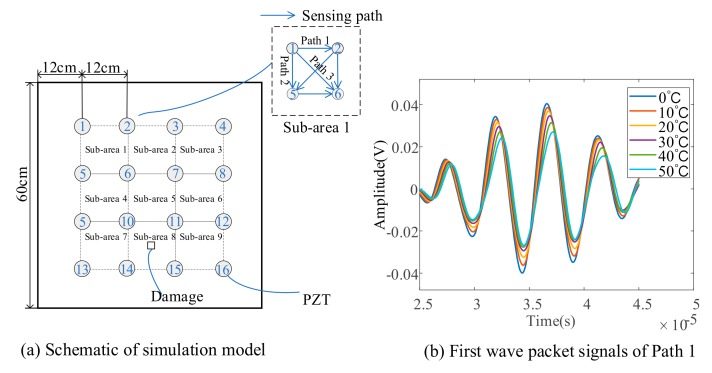
Simulation model and signals of Path 1 (PZT 1–PZT 2).

As shown in Figure 4a, a plate with dimensions 600 mm × 600 mm × 1 mm is considered as the monitored structure. Sixteen lead zirconate titanate (PZT) disks with 6.35 mm in diameter and 0.25 mm in thickness are surface-mounted on the plate. The adhesive layer thickness is 40 μm. A five-circle sinusoidal tone burst, modulated by a Hanning window with central frequency 200 kHz, is used as the exciting signal. The plate is divided into nine sub-areas each with four PZTs and six sensing paths. For example, shown in Figure 4a, sub-area 1 consists of PZT 1, 2, 5 and 6 and six paths. There are a total of 42 sensing paths.

**Figure 5:**
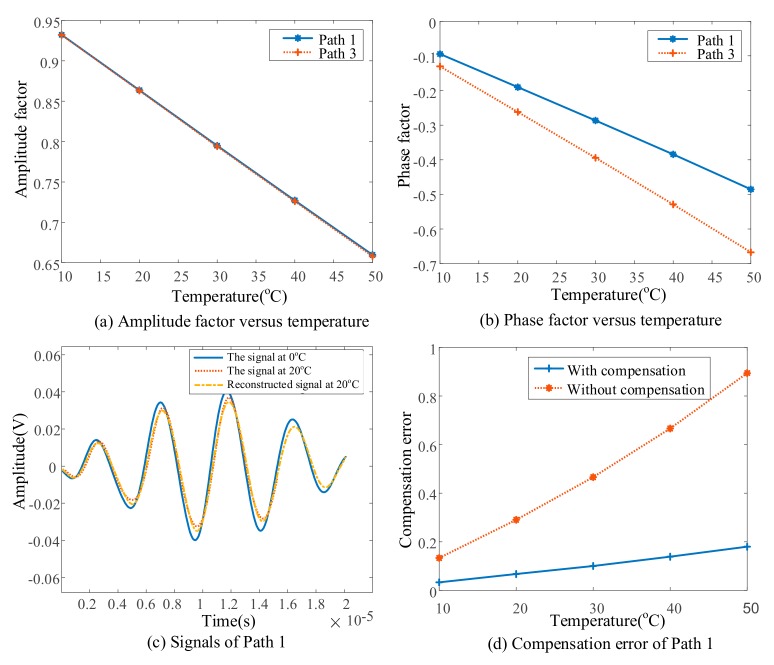
Simulation results of parameter estimation.

Six models under uniform temperature field, with temperature from 0 °C to 50 °C in steps of 10 °C, are used to calculate Lamb wave signals in the pristine structure, which are utilized to establish the data-driven model between the amplitude/phase factor and temperature. First, wave packets of signals of Path 1 (from PZT 1 to PZT 2) are shown in Figure 4b. It can be seen that as temperature increases, the overall trend of the signal is a decrease in amplitude and a delay in phase, which is the same as described by Fendzi, et al. [25]. The amplitude/phase factors with respect to the temperature of Path 1 and Path 3 (from PZT 1 to PZT 6) are shown in Figure 5a,b, from which an obvious linear trend can be observed. Therefore, a linear least regression model is utilized for data fitting and the data-driven model relating amplitude/phase factor to temperature is obtained for all exiting-sensing paths.

It can be seen from Figure 5c that the signals at 0 °C and 20 °C have an obvious difference. Another signal at 20 °C is reconstructed from the signal at 0 °C by Equation (4) based on the data-driven model. The reconstructed signal shows a good consistency with the original signal at 20 °C. A compensation error between reconstructed and original signals is introduced as

(13) $$Error=\frac{\max(abs(S_{o}-S_{r}))}{\max(abs(S_{o}))}$$

where $S_{o}$ and $S_{r}$ are original and reconstructed signals, respectively. Figure 5d gives the errors with and without compensation at different temperatures, which shows that the error with compensation between reconstructed and original signals is limited to less than 10%. This verifies the feasibility and effectiveness of the developed algorithms about parameter estimation and signal reconstruction for temperature compensation.

### 3.2. Identification of Non-Uniform Temperature Field and Damage Imaging

**Figure 6:**
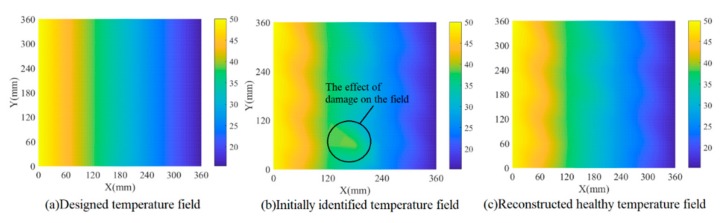
Temperature field identification results of simulation model 1.

There are three situations considered in this paper to validate the feasibility of the proposed model. As listed in Table 2, in the first simulation model, an additional through-hole damage by removing relevant elements is set at the center of Sub-area 8. The designed temperature field changes along the *x* direction from 50 °C at the left end to 15.5 °C at the right end, as shown in Figure 6a. In the second model, a damage is set at the border of Sub-area 5 and Sub-area 8 under the same temperature field as the first model. The third model contains the same damage as the first model but at a different non-uniform temperature field. Lamb wave signals from all exciting-sensing paths are acquired as the current signals. Identification and compensation of temperature field are conducted as follows, together with damage detection.

**Figure 7:**
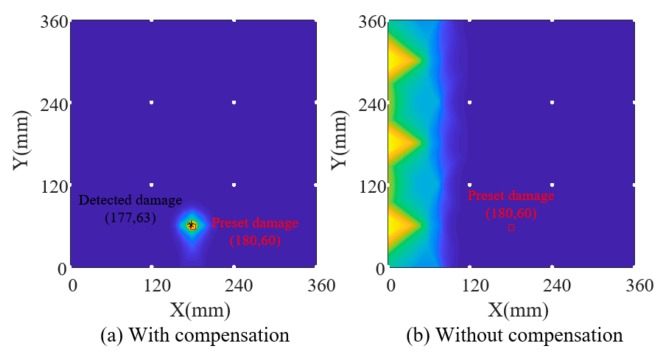
Damage identification results of simulation model 1.

**Figure 8:**
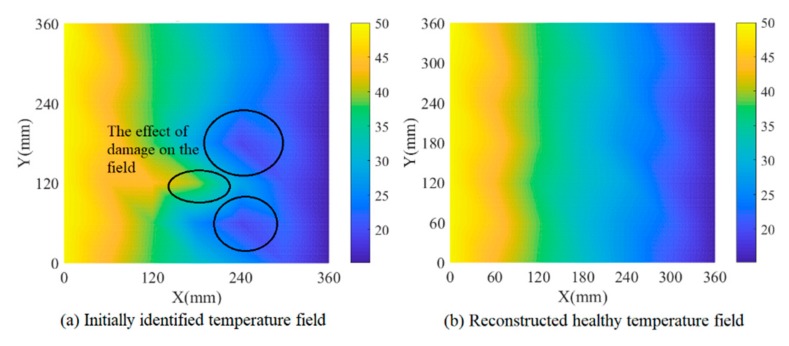
Temperature field identification results of simulation model 2.

**Figure 9:**
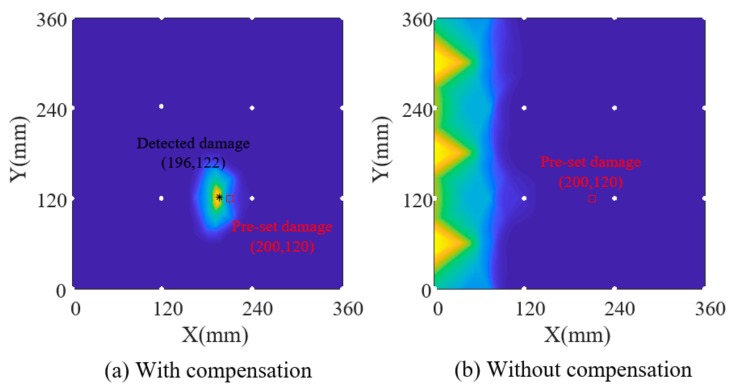
Damage identification results of simulation model 2.

Firstly, amplitude factors and phase factors of each path are extracted for the current signals by using the parameter estimation algorithm and substituted into the data-driven model, which derives the temperature for each path and initial temperature field by using interpolation. The estimated temperature of each path was considered the temperature at the midpoint of this path, and the temperatures of two cross paths were averaged. Figure 6b shows this initial structural temperature distribution, from which the slight effect of damage on the initial temperature field can be seen compared with the designed temperature in Figure 6a. Secondly, the outlier removing algorithm is applied on the initial temperature field to reconstruct the final temperature field in Figure 6c, which shows a good consistency with the designed temperature. Thirdly, based on the identified temperature field, reference signals are reconstructed according to Equation (4) in which the amplitude/phase factor can be obtained by substituting the identified temperature into the data-driven model. Finally, Lamb wave tomography is used to calculate damage probability. Figure 7a,b show damage contours with and without compensation of the first simulation model. Figure 8 and Figure 9 show the results of model 2. The white circles in the images represent PZTs. It can be seen that the damaged image after compensation apparently achieves a better effect than that without compensation in both situations.

**Figure 10:**
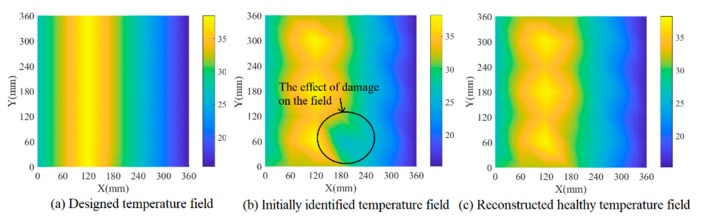
Temperature field identification results of simulation model 3.

**Figure 11:**
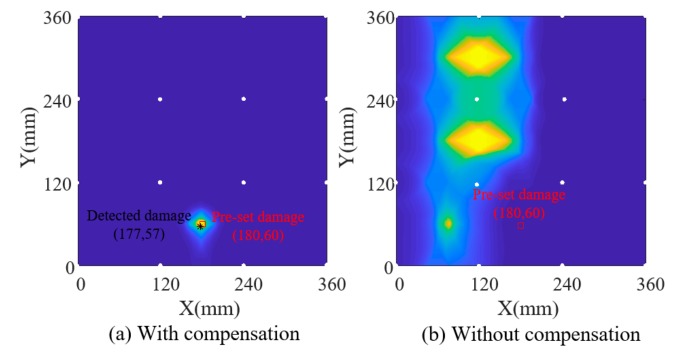
Damage identification results of simulation model 3.

In the third simulation model, the designed temperature field changes along the x direction with an increase from 27 °C to 38.5 °C first and a decrease from 38.5 °C to 15.5 °C later, which is shown in Figure 10a. The temperature identification results and damage detection results of model 3 are shown in Figure 10 and Figure 11 respectively. Comparing Figure 10b,c, we can see that the effect of damage on the field is removed after the temperature field reconstruction process. Obviously, temperature compensation makes the damage identification more reliable with an absolute error of 7.62 mm between the preset damage and the identified damage after temperature compensation.

## 4. Experiment Verification

**Figure 12:**
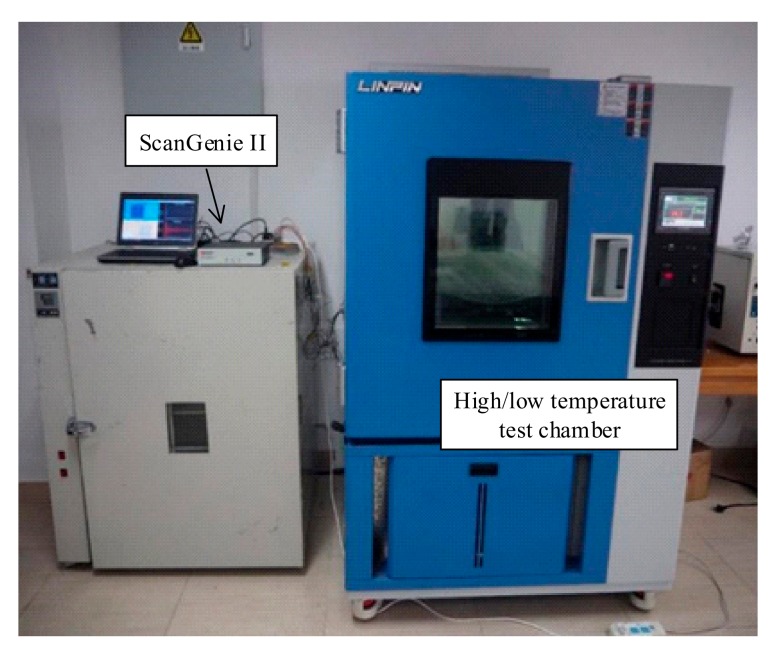
Experimental set up.

**Figure 13:**
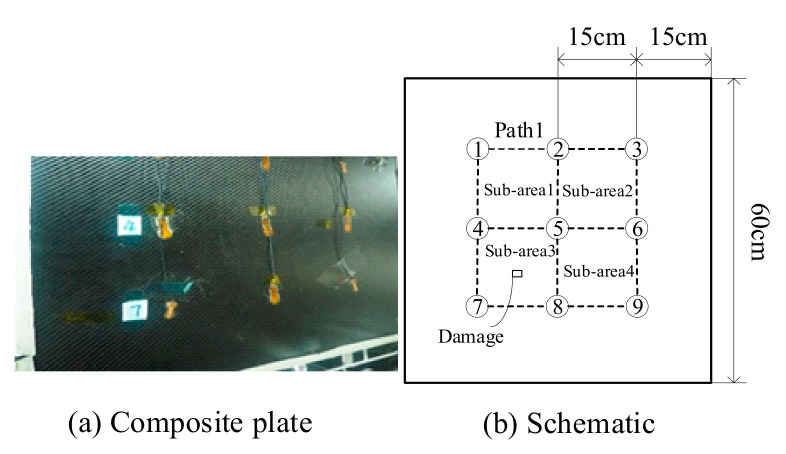
Experimental piece and its schematic.

An experiment was conducted on a composite plate with low thermal conductivity, in which the control of the non-uniform field temperature state is easier than a metal plate. Figure 12 shows details of the experimental setup. ScanGenie II made by Acellent technologies, Inc. was used for signal generation and acquisition. A high/low temperature test chamber was used to collect reference data under different temperatures from 30 °C to 70 °C in a step of 5 °C. A set of nine PZT disks with a space of 15 cm, making up four sensing areas, were used in the experiment, as shown in Figure 13a. In the experiment, the artificial damage rather than real damage was introduced with bolts affixed on the surface of structures to reduce the waste of specimen. Just as the real damages scattering Lamb wave, the geometry continuum of the structures is interrupted by bolts [7]. A five-circle sinusoidal tone burst with a center frequency of 160 kHz, modulated by Hanning window, was used as the exciting signal. Sub-area 3 and Sub-area 4, shown in Figure 13b, were locally heated to 4 °C by a heating plate to establish a non-uniform temperature field. The same temperature compensation procedure as the simulation part was applied to get the following results.

**Figure 14:**
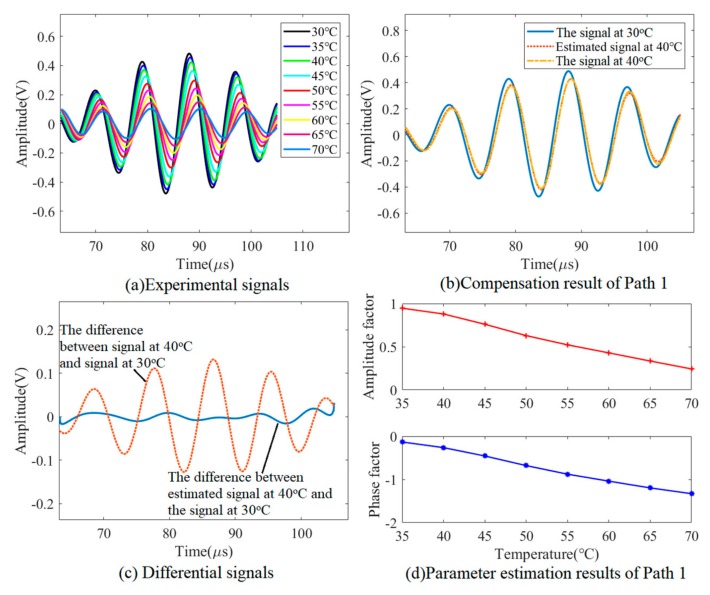
Experimental results of the parameter estimation model.

It can be seen from Figure 14a that the variation trend of the Lamb wave signals in the experiment is the same as that in the simulation, namely, a decrease in the amplitude and a delay in the phase of signals as the temperature increases. Figure 14b,c shows the results of the reference signal reconstruction of Path 1 (PZT 1–PZT 2). It can be seen directly in Figure 14c that the signal estimation procedure achieves good signal reconstruction, since the difference signal after compensation is much smaller than that before compensation. A linear relationship between amplitude/phase factor and temperature can also be observed in Figure 14d. A data-driven model is built for amplitude/phase factors versus temperature for each exciting-sensing path.

**Figure 15:**
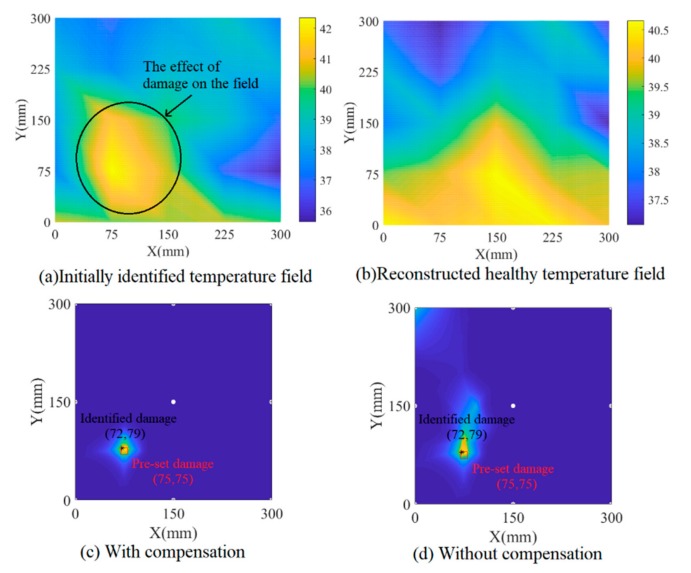
Experimental results of damage detection.

Non-uniform temperature field and damage contour are shown in Figure 15. By comparing Figure 15a with Figure 15b, the effect of the damage on the initially identified temperature field is very obvious. Figure 15c,d shows the damage identifying result with and without temperature compensation respectively. After temperature compensation, a clearer damage contour is given in Figure 15c to show the pre-set damage. It is clear that there is some virtual shadow in Figure 15d, which is caused by the temperature field. Obviously, the damage image with compensation achieves better convergence than that of without damage. The experiment demonstrates the necessity for temperature compensation for detecting small damage and the feasibility of the proposed temperature field identification and compensation method.

## 5. Conclusions

This paper introduces a non-uniform temperature field identification and compensation for Lamb wave-based damage imaging. The simulated and experimental results show that the proposed approach is effective and feasible for damage detection in large-scale aircraft structures in the actual temperature condition. The following remarks can be made based on the investigation:

 (1) The whole process of temperature compensation could be considered with six steps, including establishing a data-driven model, identifying an initial temperature field, refining the temperature field, and reconstructing the Lamb wave without damage.

 (2) Hilbert transform and LM algorithm could not only be used very well to analyze Lamb wave signals to extract the variation of amplitude/phase versus the temperature for data-driven model, but also be utilized to reconstruct Lamb wave signals at a given temperature.

 (3) The initial temperature field only has some burrs at the damage location, which can be eliminated by an outlier-removing method to get the final temperature field with good assistance with the real one.

## Figures and Tables

**Table 1:** Temperature-dependent parameters in simulation.

Parameter	The Host Structure	Piezoelectric Sensor		Adhesive
	E	d31	e33	Ea
Value at 20 °C	69 GPa	−168 × 10^−12^ m/V	15 × 10^−9^ F/m	$\begin{array}{l} 0.568T^{2}-17.5T\\ +1620MPa \end{array}$
Sensitivity	−0.04 GPa/°C	−0.5 m/V °C	0.14 × 10^−9^ F/m °C	

**Table 2:** Damage cases in the simulation.

Simulation Model	Damage Location	Temperature Field
Model 1	(240, 60)	Gradient
Model 2	(200, 120)	Gradient
Model 3	(240, 60)	More complex
